# Medical Treatment for Burn Patients with Eating Disorders: A Case Report

**DOI:** 10.1155/2011/370981

**Published:** 2011-02-23

**Authors:** Minekatsu Akimoto, Akira Takeda, Kazutaka Nagashima, Rie Uehara, Mitsuru Nemoto, Eiju Uchinuma

**Affiliations:** Department of Plastic and Aesthetic Surgery, Kitasato University School of Medicine, 1-15-1 Kitasato, Sagamihara, Minami-ku, Kanagawa 252-0374, Japan

## Abstract

There have been many cases of burn patients who also suffer from psychiatric problems, including eating disorders. We present a case of a 38-year-old female with an eating disorder and depression who became light-headed and fell, spilling boiling water from a kettle on herself at home sustaining partial thickness and full thickness burns over 5% of her total body surface area: left buttock and right thigh and calf. Eating disorders (in the present case, anorexia nervosa) cause emaciation and malnutrition, and consent for hospitalization from the patient and/or family is often difficult. During the medical treatment of burns for these patients, consideration not only of physical symptoms caused by malnutrition but also the psychiatric issues is required. Therefore, multifaceted and complex care must be given to burn patients with eating disorders.

## 1. Introduction

It is common that burn patients are often comorbid with psychiatric problems. In the United States of America, the United Kingdom, and Australia, self-inflicted burn patients account for 1% to 5% of all burn patients, and about 70% of them had psychiatric histories [1–4]. In our hospital from 1995 to 1999, 32.1% of severe burn patients suffered self-inflicted burns, and 60% of them had psychiatric histories [5]. High rates of self-inflicted burns in the Asian population have been reported [6]. The percentage of patients with psychiatric histories among the nonself-inflicted burn patients is unknown. 

When treating severe burn patients under sedation with tracheal intubation in the burn center, it is relatively not difficult to concurrently manage psychiatric disorders. During the acute stage for severe burns, systemic and wound management should be given a high priority. Psychiatric care would be required after the patient regains consciousness, after extubation, and during rehabilitation. On the other hand, in the treatment of patients with a burn covering a small area of the body that is not life threatening, concurrent psychiatric care would be beneficial during the course of the burn treatment. Sometimes, unless consent for such treatment and hospitalization is obtained, the treatment period can be prolonged because of the interruption caused by the surgical therapy.

The eating disorder, anorexia nervosa, is a disease with comorbid physical symptoms and a mental disorder due to emaciation for which the treatment is sometimes difficult even in the psychiatry department. In the case of patients with malnutrition and extreme emaciation, very often consent for treatment and hospitalization cannot be obtained from the patient or family members [7, 8]. Because inpatient treatment strategy is variable and inconsistent depending upon the medical institution, clinical practice guidelines are being made [9]. When a patient with an eating disorder sustains an accidental wound, due care should be exercised in providing treatment. Especially when treating burns, systemic management including the perisurgical period, wound management, psychiatric care, and the nutritional approach are required. Although it is difficult to treat severe burns in patients requiring tracheal intubation under sedation, who also, as we frequently encounter, have suicidal ideations or intend to commit suicide because of their depression or schizophrenia, there are other difficulties in treating burns of patients with eating disorders. We, therefore, emphasize the importance of a multidisciplinary approach for these patients. 

## 2. Case Report

**Figure 1:**
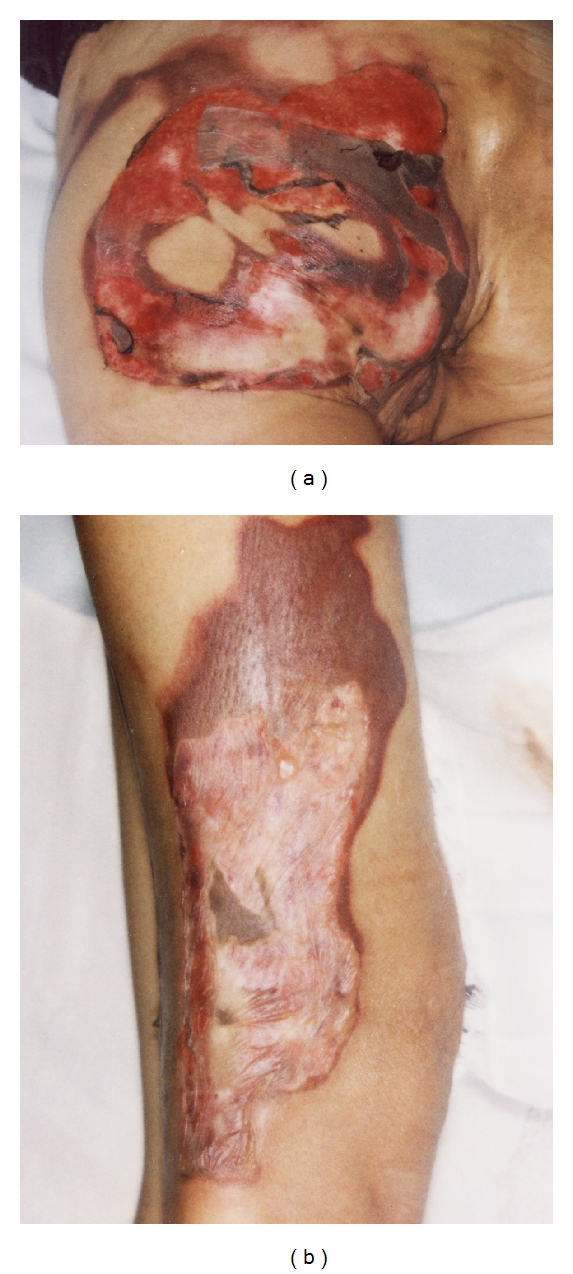
A view showing the extent of the burn: (a) the left buttock and (b) posterior right thigh, 4 days postburn.

**Figure 2:**
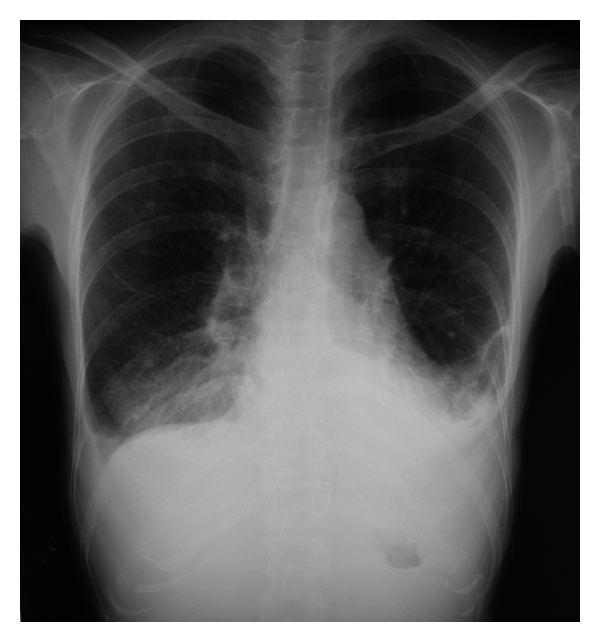
Chest X-ray showing pleural effusion 53 days postburn.

**Figure 3:**
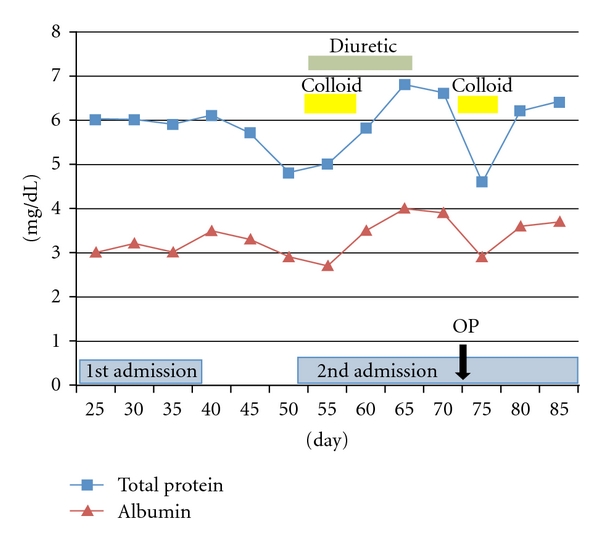
Patient's 3-month period of treatment showing protein and albumin levels.

A 38-year-old woman with an eating disorder and depression became light-headed and fell, spilling boiling water from a kettle on herself at home. She was treated for 3 days at a local medical clinic and was then referred to the Plastic and Aesthetic Surgery Department, Kitasato University Hospital, 4 days postburn. The patient sustained partial and full thickness burns over 5% of her total body surface area: the left buttock (Figure 1(a)) and the right posterior thigh and calf (Figure 1(b)). The patient was 158 cm tall with a body weight of 33 kg; thus, her BMI (body mass index) was 14. Examination of other body systems revealed that they were normal. Laboratory data results were within normal limits except hemoglobin 9.9 g/dL, total protein 6.2 g/dL, and albumin 3.0 g/dL in her blood serum. Surgical treatment and alimentation with hospitalization was planned, but consent could not be obtained. We, therefore, chose to treat her at our outpatient clinic. Consent for hospitalization was obtained 24 days postburn, and the patient was admitted. But the patient strongly desired to leave the hospital as soon as possible, so she was discharged on postburn-day 39. Burn wound infection occurred on around day 20, but the infection was controlled with silver sulfadiazine and povidone-iodine gel. On day 53, she experienced dyspnea and lower-limb edema and returned to the hospital, whereupon, pleural effusion was observed on the chest X-ray (Figure 2). Laboratory data results were: hemoglobin 7.9 g/dL, total protein 4.8 g/dL, and albumin 2.7 g/dL. The pleural effusion was treated with a colloid solution and a diuretic agent with hospitalization (Figure 3). The operation for the burn wound was performed using a sequential excision and split-thickness skin graft from the right buttock on postburn-day 76 and lasted 2 hours. The burn covered 5% of the patient's body surface area. But because of the skin graft on the left buttock and posterior right thigh, the patient required bed rest and splinting her right leg for 7 days; afterwards, the skin graft stabilized. 

**Figure 4:**
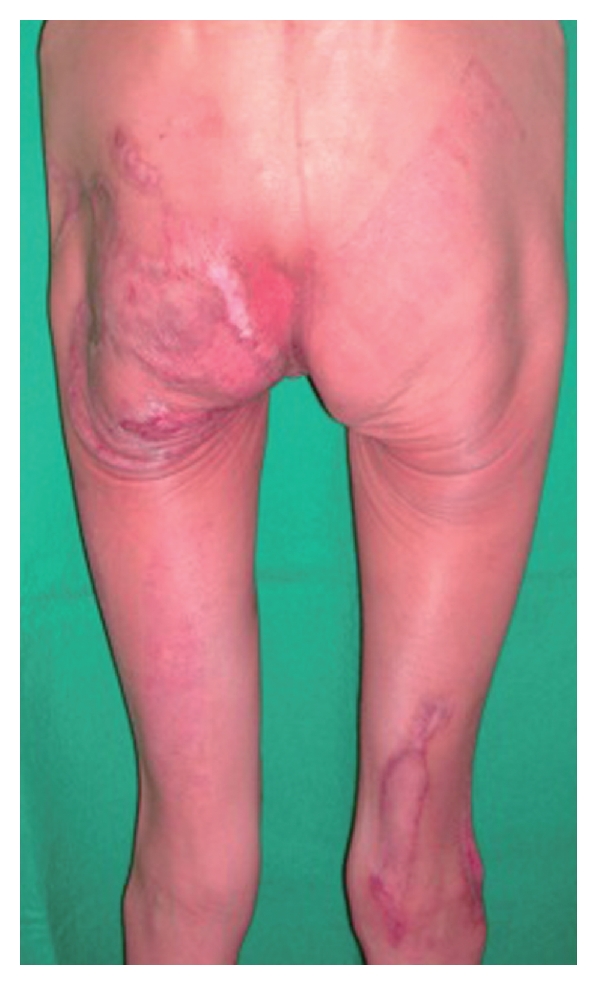
View showing the extent of the healing 8 months after surgery.

The burn wounds were successfully covered with skin grafts and treated with petroleum jelly. Rehabilitation was given for 1 week thereafter, and the patient was discharged on postburn-day 95. By postoperative-month 8, the patient was satisfied with the burn wound scars, skin grafts, and donor sites (Figure 4). 

## 3. Discussion

Medical treatment for burn patients with eating disorders most often presents some common difficulties. Consideration not only of the patient's essential burn treatment but also of the proper psychiatric treatment is usually required.

The first decision that must be made for a patient who has suffered a minor burn or a burn over a small area of the body who also suffers from an eating disorder is whether or not to hospitalize the patient for treatment or to treat the patient on an outpatient basis. For severe burn cases, such as flame burns with comorbid inhalation damage or burns accompanying other trauma, determining a treatment plan with hospitalization is relatively simple. However, if the small-area burn patient stays home during the acute phase of the injury and visits the hospital a few days later, and the patient does not have a burn-shock period but only has a burn wound, consent for hospitalization is not usually easily obtained. Standard treatment and management is provided at the burn care unit if the patient's circulatory dynamics are unstable during the acute stage of treating a burn patient with an eating disorder. However, special care is needed in a hospital ward when treating a conscious, mild-burn patient who has a comorbid psychiatric disorder. Even when consent for hospitalization can be obtained, the patient often feels uneasy regarding his or her body image or weight (emaciation) due to the eating disorder [10]. And this often results in an adverse change in the basis of the burn treatment and the patient's self-consciousness regarding the cicatrix developing, necessitating further treatment and proper adjuvant psychiatric care.

Management of a patient with a psychiatric disorder in a general hospital ward is difficult over an extended length of time. And management of trauma patients in the psychiatric ward is likewise difficult. Various ointments are regularly used for burn therapies, and often, unique treatments such as hydrotherapy are required as well. Regarding the priority and site of therapy for burn patients with eating disorders, the burn treatment would optimally be given by a burn specialist with the support of a psychiatrist in the general hospital ward. We commonly use liaison psychiatric therapy in our hospital [11].

However, there are still some remaining remedial problems. In a patient's malnutrition and low-protein state due to an eating disorder, there is severe protein and cytokine leakage from the burn wound [12, 13]. But albumin levels do not correlate with the severity of anorexia nervosa [14]. Prolonged malnutrition influences not only wound healing but also management during the perioperative period. A prolonged state of low-protein leads to pleural effusion; therefore, a colloid solution and diuretic agent are usually administered to improve respiration. 

In cases of emaciation due to an eating disorder, there is little subcutaneous and adipose tissue, and neither is sufficient immediately under the necrotic tissue resulting from a deep burn. Sufficient intra- and postoperative care must be exercised for the physical conditions of the patient due to diseased bone projection. In the present case, we used an air mat to aid in the repair of the grafted area after surgery and to help prevent decubital ulcers.

Communication with the nutrition support team regarding alimentation is necessary. The team aids in improving the patient's low-protein state. Following these measures, we have come to realize that compulsory nutrition is not necessarily an effective remedy [15, 16].

But there may not be enough time before surgery to improve the patient's nutritional state, in which event, oral administration of nutrition during burn treatment may help to decrease the patient's pain and uneasiness. In the present case, an improved nutrition plan that could be easily accepted, based on a detailed inquiry of the patient's likes and dislikes, was developed. The family cooperated with us, and the patient's favorite foods were served. The objective was for the patient to enjoy the meals as voluntarily as possible. Family support is especially important for patients with eating disorders who are concurrently receiving treatment for burn injuries, without which recovery usually takes much longer. Treatment for a nonsevere burn patient with an eating disorder is also often more difficult than that provided for a burn patient who does not have an eating disorder. In addition to the standard burn therapy and pain relief, to optimize healing and prognoses, consideration of psychiatric management during hospitalization and the perioperative period and special nutritional care with the patient's favorite foods is strongly recommended for all burn patients with eating disorders. 

## 4. Conclusions

Burn patients with eating disorders who require surgical treatment for burn wounds often refuse hospitalization. Physicians treating such patients need to take into consideration issues regarding not only wound healing but also those related to psychiatric problems, nutrition, and hospitalization.

##  Disclaimer

The authors received no funding for this work.
